# Examining the association between prior medical mistreatment and interest in and support for over-the-counter medication abortion among people seeking in-person abortion in the United States

**DOI:** 10.1186/s12913-026-14505-2

**Published:** 2026-04-13

**Authors:** Allison A. Merz-Herrala, M. Antonia Biggs, Katherine Ehrenreich, Shelly Kaller, Lauren Ralph, Karen A. Scott, Nathalie Kapp, Tammi Kromenaker, Jamila Perritt, Kari White, Daniel Grossman

**Affiliations:** 1 Department of Obstetrics, Gynecology and Reproductive Sciences, University of California San Francisco, San Francisco, CA USA; 2 Department of Obstetrics, Gynecology and Reproductive Sciences, Advancing New Standards in Reproductive Health (ANSIRH), Bixby Center for Global Reproductive Health, University of California San Francisco, Oakland, CA USA; 3 Birthing Cultural Rigor, LLC, Nashville, TN USA; 4 International Planned Parenthood Federation, London, UK; 5 Red River Women’s Clinic, Moorhead, MN USA; 6 Physicians for Reproductive Health, Washington, DC USA; 7 Resound Research for Reproductive Health, Austin, TX USA

**Keywords:** Over the counter medication abortion, Medical mistreatment, Medical discrimination, Abortion access, Person-centered care

## Abstract

**Background:**

While previous research indicates that most people seeking facility-based abortion care are interested in over-the-counter (OTC) access to medication abortion (MA), few studies have examined whether people who have experienced medical mistreatment support OTC access. We assessed whether prior experiences of medical mistreatment are associated with interest in and support for OTC MA among people seeking abortion care in the United States (US).

**Methods:**

We analyzed cross-sectional survey data from 9 US abortion facilities from June 2021 to December 2022, among people seeking in-person abortion ≥ 15 years old who spoke English or Spanish. Participants completed surveys on demographics, clinical characteristics, OTC MA interest/support, and prior experiences of medical mistreatment and their effects on subsequent care-seeking. We conducted unadjusted and adjusted regression analyses examining associations between history of medical mistreatment (none, having been ignored and/or ridiculed by healthcare providers, and having delayed or forgone care due to prior mistreatment) and interest in and support for OTC MA.

**Results:**

Among 1,360 respondents, 39% reported prior experiences of medical mistreatment, of whom 48% (258/537) had delayed care and 40% (215/537) had forgone care due to mistreatment. Overall, 72% expressed interest in and 85% supported OTC MA. In adjusted analyses, compared to people with no history of medical mistreatment, a larger proportion of participants who had delayed or forgone care (302/1,360, 22%) due to prior mistreatment expressed personal interest in (82.8%, [95% Confidence Interval 77.0%-88.6%] vs. 67.5% [61.4%-73.6%]) and support for (93.7% [89.9%-97.5%] vs. 80.5% [74.7%-86.3%]) OTC MA.

**Conclusions:**

Many patients seeking abortion care in the US have previously experienced medical mistreatment and find OTC MA appealing, especially those who have delayed or forgone healthcare due to previous mistreatment. By prioritizing patient autonomy, agency, self-efficacy and accessibility, OTC MA could improve reproductive health outcomes and equity, particularly for those historically and repeatedly marginalized by healthcare systems.

**Supplementary Information:**

The online version contains supplementary material available at 10.1186/s12913-026-14505-2.

## Background

Medication abortion (MA) involving mifepristone and misoprostol is safe and effective [1], and is now the most common abortion method in the United States (US) [2]. The shift toward MA use reflects the elimination of the in-person dispensing requirement for mifepristone, decreasing access to facility-based procedural abortion care for people living in restrictive settings, and increasing access to online and telehealth abortion provision models [3–5]. Although not approved by the US Food and Drug Administration (FDA), over-the-counter (OTC) access to MA offers a promising person-centered care model [6]. Indeed, one formulation each of oral and emergency contraception pills are now FDA-approved for OTC use, and evidence supports that their OTC availability has increased accessibility of these medications for many people [7–10]. MA meets many of the FDA criteria for OTC availability: it has a well-established safety profile, low toxicity, and low abuse potential [11, 12]. Nonetheless, mifepristone and misoprostol still require prescriptions, and only specifically certified providers can prescribe mifepristone [13]. Prior studies have demonstrated significant public and patient interest in OTC MA [14–16], and that most people understand an OTC MA prototype drug label instructions and accurately self-assess for contraindications [17–19]. Among a national sample of the general public, personal interest in OTC access has been shown to be of particular interest among people experiencing marginalization by the health care system, including those who have faced barriers accessing reproductive health care and experienced medical mistreatment [14]. However the extent to which these findings apply to the people who would be most likely to use such a product–people seeking abortion–was not explored.

OTC access to MA may benefit individuals who would prefer to avoid in-clinic services, including those who have experienced medical mistreatment. Medical mistreatment, a significant and well-documented issue among reproductive-age people assigned female at birth, may include negative healthcare experiences driven by racism and other discrimination during care provision, verbal abuse, neglect, inadequate pain management, symptom dismissal, or non-consensual care [20–24]. Prior research has demonstrated that some groups of people who are marginalized by systemic inequities disproportionately report experiences of medical mistrust and discriminatory care, including women, Black, Indigenous, and other people of color [23, 25, 26], gender expansive individuals [27], people with disabilities [28], people living on low incomes, and people whose identities or circumstances are targeted by overlapping systems of oppression [29]. Though prior studies have estimated that up to 45% of reproductive-age people assigned female at birth have experienced medical mistreatment in obstetric settings [23, 24, 30], there is limited research exploring the proportion of people reporting medical mistreatment among those seeking abortion care [14, 22]. And importantly, prior research suggests that people who have experienced medical mistreatment are more likely to consider ending their pregnancy on their own outside of the formal healthcare system, suggesting that this population might be particularly interested in OTC access to MA [22, 31]. 

OTC MA could actualize and enhance reproductive autonomy, agency, and self-efficacy, potentially mitigating barriers posed by distrust and mistrust in healthcare systems and providers. This study aims to deepen our understanding about whether previous experiences of medical mistreatment, including whether such experiences led to delayed or missed care, are associated with abortion patients’ interest in and support for OTC MA.

## Methods

### Study design

This study analyzes data collected as part of a study comparing abortion patients’ standard MA eligibility assessment by a clinician to MA eligibility self-assessment using a self-administered survey [19, 32]. The survey also included questions about past medical mistreatment experiences and attitudes toward OTC MA. We recruited participants from 9 US abortion facilities in Arizona, California, Florida, Illinois, Iowa, North Dakota, Pennsylvania, and Tennessee between June 2021 and December 2022. Sites were selected for regional diversity and their ability to accommodate survey completion before ultrasound. Eligibility criteria included being 15 years or older, English or Spanish proficiency, and seeking medication or procedural abortion. Participants were informed that the study would involve completing a 10-minute survey asking about their pregnancy, medical history, and socioeconomic background. The participant survey was developed with input from an advisory board of reproductive health and justice leaders and clinicians, many of whom represent the lived experiences of people who lack equitable access to abortion. Potential participants first completed an electronic eligibility survey. Those interested and eligible consented and completed an electronic survey in English or Spanish, before ultrasound, about their pregnancy, medical history, background characteristics, and history of experiences of medical mistreatment, interest and support for OTC MA model. We gave participants a $5 gift card for completing the eligibility survey, and a $25 gift card for completing the survey.

### Outcome variables

Our two primary outcomes included *personal interest in* and *support for* OTC access using questions developed based on previous research [15, 16]. The survey first presented participants with a description of a theoretical OTC MA model (Box 1, Supplement), followed by two questions to gauge: (1) personal interest in OTC MA, “Would you be personally interested in buying abortion pills without a prescription from a drug store, pharmacy or grocery store for yourself?” (Definitely yes, Probably yes, Probably no, Definitely no, and Not sure); and (2) support for OTC MA, “Even if you are not interested in this option for yourself, would you be in favor of other pregnant people being able to buy abortion pills in a drug store without a prescription?” (In favor, Somewhat in favor, Somewhat opposed, Opposed, and Don’t know). For analyses, we dichotomized answer options to “Probably/Definitely yes” vs “Probably/Definitely no or Don’t know” and “In favor/Somewhat in favor” vs “Somewhat opposed/Opposed or Not sure.” We also asked people to select advantages and disadvantages of an OTC model from a list, including an open-ended “other” category. The pre-defined listed advantages and disadvantages were selected based on previous quantitative and qualitative research [16, 33]. 

### Independent variables

Our primary independent variables were based on five survey questions assessing medical mistreatment history experienced prior to the abortion visit. These medical mistreatment items were developed by our group and have been found to be associated with views about criminalizing and considering ending a pregnancy on one’s own outside of the formal healthcare system [22, 34, 35] and interest in OTC MA [14], and to be experienced more often by people living with a disability [36]. 

In line with prior research by our group [14, 22], we asked participants: “When seeking health care, (1) “have you *ever felt ignored* by doctors, nurses, or other providers?; (2) “have you *ever felt ridiculed or humiliated* by doctors, nurses, or other providers?”; and (3) whether providers had ever made you feel your “*symptoms were not real*,* not severe or not important*?” Participants who answered “Yes” to any of these questions were asked whether it was true for them that: (4) “*These interactions* between me and doctors, nurses, or other providers *have caused my medical care to be delayed*”; and (5) “I have *not gotten the medical care I needed because of these interactions*.” All mistreatment questions had “Yes”, “No”, and “Not sure” response options.

For the analyses assessing associations between prior medical mistreatment and OTC MA attitudes, we dichotomized all mistreatment items by collapsing “No” and “Not sure” responses. We created a composite 4-category variable representing medical mistreatment: (1) “No mistreatment”, (2) “Symptoms ignored” (which included reports of “ever felt ignored” by providers and/or had been made to feel their “symptoms were not real, not severe or not important”, but no reports of ridicule or humiliation and no delayed or missed care due to these interactions), (3) “Felt ridiculed or humiliated” (may have also had symptoms ignored, but no delayed or missed care due to these interactions), and (4) “Delayed or missed care due to negative interactions.” The questions characterizing prior experiences of medical mistreatment and this four-part categorization of those experiences align with prior research by our group [14, 36]. 

### Covariates

We selected additional model covariates a priori, based on known factors associated with abortion attitudes [37], including: age group, race/ethnicity, whether the participant was born in the US, highest education level (high school or less vs. any college or more), food/housing insecurity (either/both vs. none), urban vs. rural community, paying out of pocket for abortion care, self-reported pregnancy duration (based on the question “How many weeks pregnant do you think you are today?”), abortion history, and preferred abortion type. We also included an abortion policy context variable (supportive, middle ground, hostile, and extremely hostile) based on the participants’ state of residence, according to April 2022 (mid-point in the study) Guttmacher Institute data [38]. 

### Statistical analysis

We estimated frequencies for participant characteristics, prior medical mistreatment, interest in and support for OTC MA, and its perceived advantages and disadvantages. We used bivariable and multilevel mixed effects logistic regression models to estimate the marginal predicted probabilities of interest in and support for OTC MA by medical mistreatment and model covariates. Observations with missing data on any variables included in multivariable models were excluded. We conducted analyses in Stata SE 18.0. We report significance at *p* ≤ 0.05. We used the STROBE checklist to ensure comprehensive and transparent reporting of observational study methods and findings [39]. 

### Sensitivity analysis

To discern whether disaggregating respondents who answered “Not sure” from those who answered “No” to the questions about prior medical mistreatment experiences would change the outcomes from the analyses, we conducted a sensitivity analysis by running the univariate and multilevel mixed effects logistic regression models with the “No” and “Not sure” respondents disaggregated.

## Results

Among 2,846 people approached, 1,775 (62%) were interested, 1,591 were eligible, and 1,386 consented to participate and had complete clinician data. We only included participants with complete clinician data which was used to fill in missing demographic and gestational duration data and resolve inconsistencies. We excluded participants who didn’t answer any medical mistreatment, OTC MA interest, or OTC MA support questions (*n* = 26), leaving a final analytical sample of 1,360 participants. The percentage of total participants enrolled by individual clinic site ranged from 4 to 22%. Participant characteristics are presented in Table 1. The mean age was 27 (SD 6). Most participants identified as either Non-Hispanic Black (32.7%), Non-Hispanic White (29.0%) or Hispanic/Latinx (28.7%). Nearly half reported food or housing insecurity (49.4%). Most paid out of pocket for their abortion (67.9%), lived in states with extremely hostile abortion policies (61.0%), were ≤10 weeks pregnant (75.8%), and did not report a previous abortion (54.8%).

**Table 1:** Characteristics of patients presenting for abortion care at 9 facilities in 8 US states from June 2021 to December 2022 (*N* = 1,360)

Variable	Total, *n* (%)
**Age in years, mean (SD)**	**27 (6)**
15–17	13 (1.0)
18–19	102 (7.5)
20–24	461 (33.9)
25–29	386 (28.4)
30–34	236 (17.4)
35–46	162 (11.9)
**Race and Ethnicity**	
Black or African American (Non-Hispanic (NH))	444 (32.7)
White (NH)	394 (29.0)
Hispanic/Latinx	390 (28.7)
Asian/Pacific Islander (NH)	26 (1.9)
Asian	25 (1.8)
Native Hawaiian/Pacific Islander	1 (0.1)
American Indian or Native American (NH)	23 (1.7)
Multi-Racial/Other	79 (5.8)
Multi-racial	69 (5.1)
Middle Eastern or North African (NH)	3 (0.2)
Other	7 (0.5)
Missing	4 (0.3)
**Born in the United States**	
Yes	1,233 (90.7)
No	124 (9.1)
Missing	3 (0.2)
**Educational Attainment**	
High school diploma/GED* or less	544 (40.0)
Any college or more	814 (59.9)
Missing	2 (0.1)
**Food/Housing insecurity in past year**	
Yes	672 (49.4)
No/Not sure	685 (50.4)
Missing	3 (0.2)
**Paying out of pocket for this abortion**	
Yes	924 (67.9)
No	436 (32.1)
**Abortion policy in state of residence** ^†^	
Supportive	271 (19.9)
Middle-ground	27 (2.0)
Hostile	118 (8.7)
Extremely Hostile	829 (61.0)
State of residence not reported	115 (8.5)
**Local Community**	
Large city	635 (46.7)
Suburb near large city	231 (17.0)
Small city or town	386 (28.4)
Rural area	59 (4.3)
Don’t know	49 (3.6)
**Gestational duration**,** based on self-reported weeks pregnant**^**‡**^	
≤10 weeks	1,031 (75.8)
>10 weeks	255 (18.8)
Not sure	74 (5.4)
**Prior abortion experience**	
No prior abortion	745 (54.8)
Prior medication abortion (MA) (may have also had procedural abortion)	324 (23.8)
Prior procedural abortion, no MA	291 (21.4)
**What type of abortion do you prefer to have?**	
Somewhat/Strongly prefer procedural abortion	380 (27.9)
Somewhat/Strongly prefer MA	770 (56.6)
No preference/Don’t know	210 (15.5)

*General Educational Development, certifying academic knowledge equivalent to a high school diploma. ^†^Policy as of 04/2022 based on data from the Guttmacher Institute [38]. ^**‡**^Based on the survey question “How many weeks pregnant do you think you are today?”

**Fig. 1:**
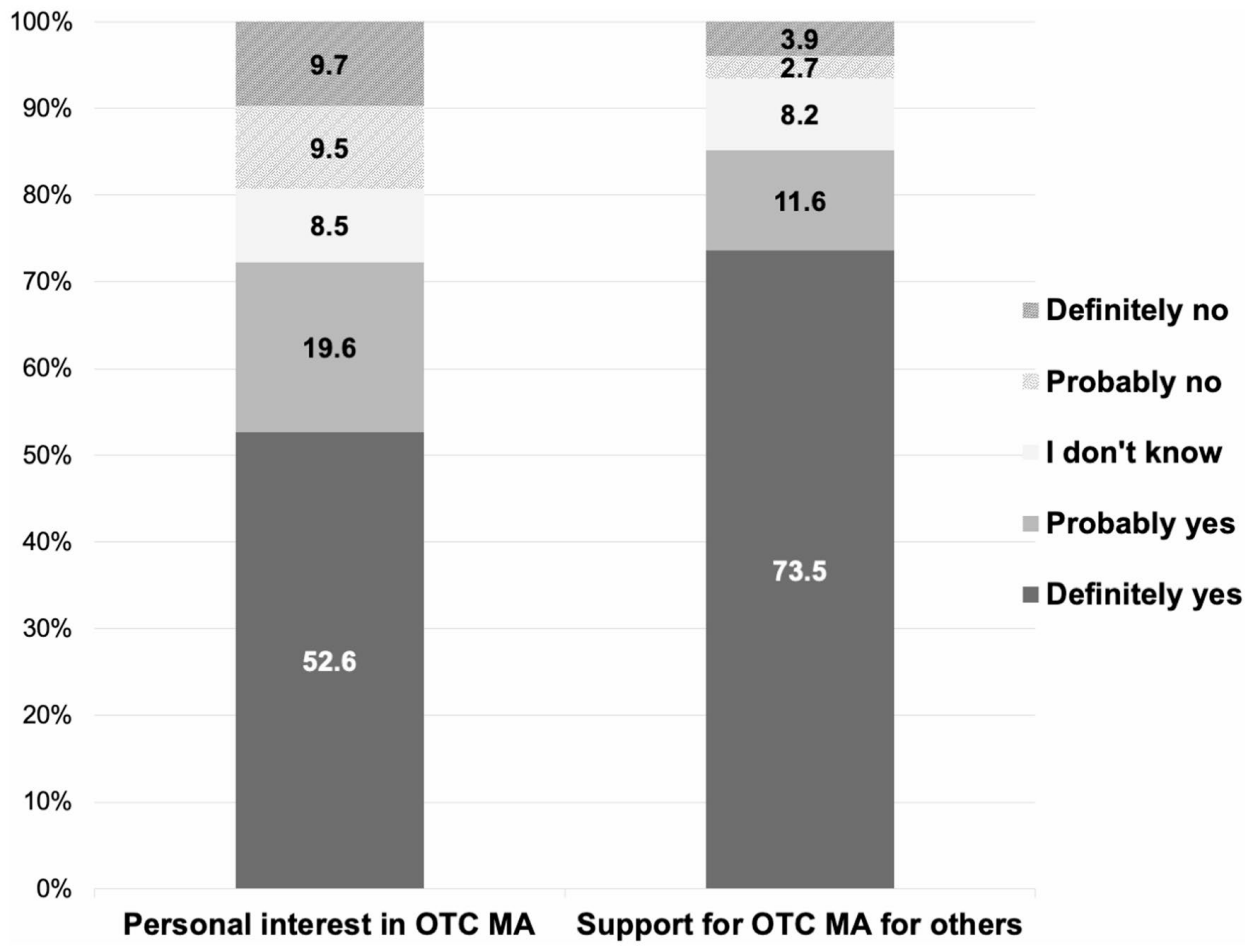
Interest in and Support for over-the-counter (OTC) access to medication abortion (MA) among people presenting for abortion care at 9 abortion facilities in 8 US states from June 2021 to December 2022 (*N* = 1,360). Stacked bars show the percentage distribution of responses to ‘Personal interest in OTC MA’ and ‘Support for OTC MA for others’ questions. Percentages sum to 100% within each question. *n* = 1,357 for the ‘Support for Others’ question as there were 3 missing responses to that question

Participants’ personal interest in and support for OTC MA are shown in Fig. 1. Most people indicated that they would definitely (52.6%) or probably (19.6%) be interested in OTC MA and would be in favor (73.5%) or somewhat in favor (11.6%) of OTC MA access for others. Perceived advantages and disadvantages of OTC MA, all selected from a pre-defined list included in the survey, are presented in Table 2. The most common perceived advantages included earlier abortion access (80.1%), privacy (70.2%) and convenience (68.5%). The most common perceived disadvantages included concerns that people might take the pills incorrectly (56.8%) and might be forced to take pills without consent (50.4%). 50 participants (3.7%) saw no advantages of the OTC model, whereas 125 (9.2%) saw no disadvantages.

**Table 2:** Perceived advantages and disadvantages of over-the-counter (OTC) access to medication abortion (MA) among people presenting for abortion care at 9 abortion facilities in 8 US states from June 2021 to December 2022 (*N* = 1,360)

Variable	Total (*n*,%)
Perceived advantages of OTC MA*	
Help people get abortion earlier in pregnancy	1,089 (80.1)
Could be more private	954 (70.2)
Could be more convenient	931 (68.5)
Could be less expensive	846 (62.2)
Could avoid going to a clinic	745 (54.8)
Could avoid having to see a doctor or nurse	478 (35.2)
Could be more effective	298 (21.9)
Could be safer	283 (20.8)
Don’t see any advantages	50 (3.7)
Perceived disadvantages of OTC MA*	
People might take pills incorrectly	772 (56.8)
Might be forced to take pills without consent	686 (50.4)
Questions may be unanswered before abortion	562 (41.3)
People might not see doctor/nurse before abortion	519 (38.2)
Could be less effective	293 (21.5)
Someone could find pills at home	269 (19.8)
Could be too convenient	210 (15.4)
Could be more expensive	196 (14.4)
Could be less safe	160 (11.8)
Don’t see any disadvantages	125 (9.2)

*Perceived advantages and disadvantages of an OTC model were selected by respondents from a pre-defined list which was developed based on previous quantitative and qualitative research [16, 33].

Table 3 shows the proportions of participants reporting prior medical mistreatment by health care providers. Of 1,360 respondents, 39.5% reported any of the three forms of prior medical mistreatment. More specifically, 31.1% of 1,360 respondents reported having felt ignored, 31.7% were made to feel their symptoms were not real, not severe, or not important, and 21.3% had felt ridiculed or humiliated by providers; 19.0% had delayed medical care, and 15.8% had not gotten needed care due to these prior negative interactions.

**Table 3:** Experiences of medical mistreatment and interest in and support for OTC MA among people presenting for abortion care at 9 abortion facilities in 8 US states from June 2021 to December 2022 (*N* = 1,360)

Variable	Total	Definitely or probably interested in OTC MA (*n*, %)	In favor or somewhat in favor of OTC MA* (*n*, %)
**Total**	**1,360**	**983 (72.3)**	**1,157 (85.1)**
**Medical mistreatment** ^^^			
Any mistreatment	537	429 (79.9)	493 (91.8)
When seeking health care, ever felt:			
ignored by health care providers			
No	858	590 (68.8)	704 (82.1)
Not sure	77	53 (68.8)	59 (76.6)
Yes	424	339 (80.0)	393 (92.7)
Missing	1	1 (100)	1 (100)
health care providers made you feel your symptoms were not real, not severe, or not important			
No	879	604 (68.7)	718 (81.7)
Not sure	47	29 (61.7)	35 (74.5)
Yes	431	348 (80.7)	401 (93.0)
Missing	3	2 (66.7)	3 (100)
ridiculed or humiliated by health care providers			
No	1,014	704 (69.4)	841 (82.9)
Not sure	56	38 (67.9)	43 (76.8)
Yes	290	241 (83.1)	273 (94.1)
**Medical mistreatment led to delaying or forgoing care**			
These interactions caused my medical care to be delayed			
No prior medical mistreatment	823	554 (67.3)	664 (80.7)
No	218	160 (73.4)	193 (88.5)
Not sure	60	45 (75.0)	53 (88.3)
Yes	258	223 (86.4)	246 (95.3)
Missing	1	1 (100)	1 (100)
I have not gotten the medical care I needed because of these interactions			
No prior medical mistreatment	823	554 (67.3)	664 (80.7)
No	258	195 (75.6)	229 (88.8)
Not sure	62	51 (85.3)	55 (88.7)
Yes	215	181 (84.2)	207 (96.3)
Missing	2	2 (100)	2 (100)

ᶺAny mistreatment=“Yes” answer to at least one of the three medical mistreatment questions; *3 respondents did not answer the question assessing support for OTC MA for others

In unadjusted analyses, people who had previously experienced ridicule by healthcare providers (90.2%, [95% confidence interval (CI) 83.4%-97.0%], *p* = 0.02) or had delayed or missed care due to mistreatment (94.6%, [95% CI 91.2%-97.9%], *p* < 0.001) were more likely to support OTC access than people who had no prior medical mistreatment (79.8%, [95% CI 73.5%-86.0%]) (Table 4). People who had delayed or missed care due to mistreatment were more likely to report personal interest in OTC access (82.6%, [95% CI 75.7%-89.4%]) than those reporting no prior medical mistreatment (65.9%, [95% CI 57.6%-74.3%], *p* < 0.001). In adjusted analyses, those who had delayed or missed care due to medical mistreatment were more likely to report personal interest in (82.8%, [95% CI 77.0%-88.6%] vs. 67.5%, [95% CI 61.4%-73.6%], *p* < 0.001) and support for (93.7%, [95% CI 89.9%-97.5%] vs. 80.5%, [95% CI 74.7%-86.3%], *p* < 0.001) OTC MA compared to those with no history of mistreatment. Supplementary Table S1 shows all covariates included in the multivariable model investigating the associations between prior mistreatment and OTC attitudes. The sensitivity analysis re-estimating the multilevel mixed-effects logistic regression models with ‘No’ and ‘Not sure’ responses modeled as separate categories yielded substantively similar results (Supplementary Table S2).

**Table 4:** Bivariable and multivariable analyses examining the association between prior history of medical mistreatment and personal interest in and support for over-the-counter (OTC) access to medication abortion (MA) among people presenting for abortion care at 9 abortion facilities in 8 US states from June 2021 to December 2022

	Personal Interest in OTC MA			Support for OTC MA for others		
	Marginal predicted probability	95% Conf. Interval	Pᶺ	Marginal predicted probability	95% Conf. Interval	Pᶺ
Unadjusted*						
**Overall predicted probability**	70.5%	63.2%-77.9%		84.4%	79.5%-89.3%	
Prior history of medical mistreatment						
None	65.9%	57.6%-74.3%	Ref.	79.8%	73.5%-86.0%	Ref.
Ignored, not ridiculed, no delayed or missed care	68.1%	57.5%-78.7%	0.60	85.9%	78.8%-93.1%	0.09
Ridiculed (may or may not have also been ignored), no delayed or missed care	75.7%	65.0%-86.5%	0.06	**90.2%**	**83.4%-97.0%**	**0.02**
Delayed or missed care due to prior mistreatment	**82.6%**	**75.7%-89.4%**	**< 0.001**	**94.6%**	**91.2%-97.9%**	**< 0.001**
**Adjusted** ^**‡**^						
**Overall predicted probability**	71.3%	66.0%-76.7%		84.2%	79.4%-88.9%	
Prior history of medical mistreatment						
None	67.5%	61.4%-73.6%	Ref.	80.5%	74.7%-86.3%	Ref.
Ignored, not ridiculed, no delayed or missed care	68.1%	59.1%-77.1%	0.89	85.6%	78.5%-92.7%	0.14
Ridiculed (may or may not have also been ignored), no delayed or missed care	74.6%	64.7%-84.5%	0.17	88.5%	80.9%-96.1%	0.08
Delayed or missed care due to prior mistreatment	**82.8%**	**77.0%-88.6%**	**< 0.001**	**93.7%**	**89.9%-97.5%**	**< 0.001**

ᶺP values are based on multilevel mixed effects logistic regression which accounted for clustering by recruitment site; *Unadjusted analysis: *N* = 1,357 for “Personal interest” outcome, *N* = 1,354 for “Support for others” outcome; ^**‡**^Adjusted analysis: *N* = 1,345 for “Personal interest” outcome, *N* = 1,342 for “Support for others” outcome. Covariates include Race/Ethnicity, Age group, US born, Highest level of education, Food or housing insecurity, Paying out of pocket for abortion, Abortion policy in state of residence, Local community, History of abortion, and Abortion preference; **Bold** indicates statistical significance at *p ≤ *0.05

## Discussion

We surveyed a diverse sample of individuals seeking facility-based abortion care around the time that the Dobbs decision eliminated the federal right to abortion in the US, and found that many people seeking abortion care in the US had previously experienced medical mistreatment (39%) and that delaying or forgoing care due to prior mistreatment was associated with greater support for and interest in OTC MA access. Though prior research has demonstrated an association between prior medical mistreatment and support for and personal interest in accessing OTC MA among a nationally-representative sample in the US [14], this study adds new evidence examining this association among people accessing abortion care, the population most likely to use OTC MA. Furthermore, this study adds novel evidence regarding relationships between delaying or forgoing care due to past medical mistreatment and OTC MA attitudes.

Broadly, we observed positive attitudes toward OTC MA across participant characteristics: substantial majorities expressed personal interest in (72.3%) and support for (85.1%) OTC MA. These proportions are somewhat higher than those reported in prior studies of abortion patients from 2017 to 2020 which found 63%-65% of participants were interested in and 72%-83% supportive of OTC MA [15, 40]. This increase in personal interest in and support for OTC MA over time has also been found among the general population in the US [14], and may reflect a reaction to the peeling back of abortion rights following the Dobbs decision and increased public awareness of the negative ramifications of reduced abortion access for pregnant people and families.

In our adjusted analyses, a history of delaying or forgoing care due to prior medical mistreatment was significantly associated with greater personal interest in and support for OTC MA, suggesting that negative healthcare experiences may influence preferences for care models that are less dependent on provider-patient interactions. This finding is in line with prior research demonstrating that past negative healthcare experiences can lead to fear of the healthcare system and delays in seeking healthcare [41, 42], and that people who have experienced mistreatment would consider self-managing their abortion [22]. Additionally, given the substantial proportion of participants in our study reporting prior experiences of medical mistreatment, which is in line with rates reported in prior studies [14, 22–24], the increased interest in OTC MA in this population could have important implications for the demand for OTC MA access, if it were to become available. These findings also highlight a need to better address medical mistreatment by providers, which is impacting patients’ care-seeking.

OTC MA advantages endorsed by participants reflect the importance of timely access, confidentiality, and ease of use. Conversely, participants voiced concerns regarding OTC MA including risks of incorrect pill usage, coercion, having unanswered questions prior to abortion, and lack of clinician involvement. These concerns are similar to those identified in prior studies [15, 16, 40] and underscore the need for education about MA safety and to explore supportive services to ensure safe, informed OTC MA use. Addressing potential disadvantages through clear instructions and accessible helplines will be crucial to OTC implementation.

While it is unlikely that an OTC MA product would become available in pharmacies in states that restrict or ban abortion, this model of care still has the potential to increase MA access for people living in restrictive states, as they could possibly travel to another state to obtain MA medications OTC. It bears stating that having to cross state lines to access abortion care is ethically indefensible, but is a reality that many people in the US face today. For both those forced to travel across state lines and for those obtaining care within their state of residence, OTC MA could offer advantages over telehealth models: it would not require a mailing address or internet access, could facilitate distribution through social networks, and it might allow for receipt by mail, though the legal status of mail distribution is currently unclear.

Our study has strengths and limitations. It has a large geographically diverse sample. These data add to the literature assessing associations between prior medical mistreatment and OTC MA attitudes among patients seeking abortion care. Limitations include that we did not ask details of when, where, or how often people may have been mistreated within healthcare settings, and the specifics of past mistreatment experiences could impact abortion care attitudes. For example, mistreatment in a primary care setting versus a family planning setting might differentially impact support for OTC access. Similarly, a remote history of mistreatment might have less impact on OTC attitudes compared to more recent mistreatment or a repeated history of mistreatment. Our sample represents diverse identities, including many who have historically been marginalized or discriminated against by the healthcare system. While most people in our sample reported BIPOC identities and about half reported recent experiences of food or housing insecurity and paying out of pocket for their care, there is a need for future research on OTC MA among other populations who historically have been discriminated against by the health care system, such as people living with disabilities and sexual and gender minorities. While our 5-item measure has not been compared to other formal measures of mistreatment, our items align closely with the core domains of these measures [43, 44]. This is an area for future research. Our study population does not represent all people seeking abortion care in the US. By design, we only included people accessing facility-based abortion care, therefore potentially missing those who prefer telehealth or self-managed abortion, a population that might be particularly interested in and benefit from an OTC model. There were many missing responses to the state of residence question (8.5%), perhaps suggesting that some respondents feared disclosing their state of residence due to concern for criminalization for seeking abortion care.

This study highlights several areas where more research is needed. Further research on OTC MA safety and efficacy will need to be submitted to the FDA as part of a prescription-to-OTC switch application. Ensuring that OTC MA is accompanied by adequate support, education, and safeguards will be essential to maximize its benefits and minimize risks and barriers, particularly considering the interest in OTC MA among those mistreated by the healthcare system. This is particularly important considering that despite OTC availability of oral and emergency contraception, their accessibility and the person-centeredness of their use when accessed OTC have been compromised by persistent misinformation and access barriers including cost, age restrictions, and variable pharmacy stocking and dispensing [9, 45–47]. Dedicated study among people who have experienced medical mistreatment could help inform how to create accessible and trustworthy support systems for people using MA, including both OTC and prescribed MA. Given the high incidence of medical mistreatment and significant knowledge gaps among clinicians around sexual and reproductive health preferences and experiences of people with disabilities [48], future studies on OTC MA among this population are needed.

## Conclusions

Our findings demonstrate strong interest in and support for OTC MA among people seeking abortion care, especially among those who have experienced medical mistreatment. Given people’s varied preferences for abortion care, OTC MA should exist as part of a robust abortion care ecosystem where everyone is able to access the type of abortion care they want or need. This study highlights the reality that many people seeking abortion have experienced medical mistreatment, which may exacerbate existing barriers to care since many people’s prior mistreatment caused them to delay or forgo needed medical care. OTC MA could improve reproductive health outcomes and equity, particularly for those historically and repeatedly marginalized by healthcare systems, by prioritizing patient autonomy, agency, self-efficacy and accessibility. Policymakers, healthcare providers, and reproductive focused bioethicists and implementation scientists should consider these preferences and the potential benefits of OTC MA in enhancing reproductive autonomy and addressing healthcare inequities.

## Supplementary Information

Below is the link to the electronic supplementary material.

Supplementary Material 1

## Data Availability

Study data are not publicly available due to their sensitive nature and because it could pose a risk to the privacy of participants. The data include information about people’s health behaviors, including abortion, which is a sensitive, stigmatized, and sometimes criminalized behavior. Researchers with a legitimate interest in replicating our findings or conducting secondary analyses can contact the corresponding author to discuss the possibility of obtaining anonymized data or aggregate statistics. We will consider all requests on a case-by-case basis and prioritize those that align with the original research objectives and participant consent.

## Abbreviations

OTC: Over-the-counter
MA: Medication abortion
US: United States
FDA: Food and Drug Administration
