# Supplementary material for: Examining the association between prior medical mistreatment and interest in and support for over-the-counter medication abortion among people seeking in-person abortion in the United States
Source: BMC Health Serv Res. 2026 Apr 13;26:736. doi: 10.1186/s12913-026-14505-2 (PMC13195937; doi:10.1186/s12913-026-14505-2)
Supplement: Supplementary file 1 — Supplementary Material 1 [file 12913_2026_14505_MOESM1_ESM.docx]

**Supplement**

**Box 1: Description of theoretical OTC MA model**

| Medication abortion, or the abortion pill, is a safe and effective way to end an early pregnancy and is available from a doctor’s office or clinic. In the future, it may be possible to get abortion pills without a prescription from a drug store, pharmacy, or grocery, just like condoms or pregnancy tests. You would receive detailed information about how to take the pills and could talk to the pharmacist at the store or call a 24-hour telephone number to answer your questions. Although most people can tell on their own if the abortion is successful, you could go to a clinic afterwards to make sure the medication worked. If you preferred, you could still get the pills from a clinic. When answering the questions below, do not focus on cost because it is too early to know the cost of this option. This is an idea of something that is not currently happening. We want to know what you think about it. |
| --- |

**Table S1:** Bivariable and multivariable analyses examining the association between prior history of medical mistreatment and personal interest in and support for over-the-counter (OTC) access to medication abortion among people presenting for abortion care at 9 abortion facilities in 8 U.S. states from June 2021 to December 2022, including all model covariates (N=1,360)

|  | **Personal Interest in OTC MA** | | | **Support for OTC MA for others** | | |
| --- | --- | --- | --- | --- | --- | --- |
|  | **Margin** | **95% Conf. Interval** | **Pᶺ** | **Margin** | **95% Conf.**  **Interval** | **Pᶺ** |
| **Unadjusted*** | | | | | | |
| **Overall predicted probability** | 70.5% | 63.2%-77.9% |  | 84.4% | 79.5%-89.3% |  |
| Prior history of medical mistreatment | | | | | | |
| None | 65.9% | 57.6%-74.3% | Ref. | 79.8% | 73.5%-86.0% | Ref. |
| Ignored, not ridiculed, no delayed or missed care | 68.1% | 57.5%-78.7% | 0.60 | 85.9% | 78.8%-93.1% | 0.09 |
| Ridiculed (may or may not have also been ignored), no delayed or missed care | 75.7% | 65.0%-86.5% | 0.06 | **90.2%** | **83.4%-97.0%** | **0.02** |
| Delayed or missed care due to prior mistreatment | **82.6%** | **75.7%-89.4%** | **<.001** | **94.6%** | **91.2%-97.9%** | **<.001** |
| **Adjusted^‡^** | | | | | | |
| **Overall predicted probability** | 71.3% | 66.0%-76.7% |  | 84.2% | 79.4%-88.9% |  |
| Prior history of medical mistreatment | | | | | | |
| None | 67.5% | 61.4%-73.6% | Ref. | 80.5% | 74.7%-86.3% | Ref. |
| Ignored, not ridiculed, no delayed or missed care | 68.1% | 59.1%-77.1% | 0.89 | 85.6% | 78.5%-92.7% | 0.14 |
| Ridiculed (may or may not have also been ignored), no delayed or missed care | 74.6% | 64.7%-84.5% | 0.17 | 88.5% | 80.9%-96.1% | 0.08 |
| Delayed or missed care due to prior mistreatment | **82.8%** | **77.0%-88.6%** | **<.001** | **93.7%** | **89.9%-97.5%** | **<.001** |
| **Covariates** | | | | | | |
| Racial/Ethnic Identity | | | | | | |
| Black or African American (Non-Hispanic (NH)) | 67.4% | 60.5%-74.4% | 0.68 | 79.1% | 72.5%-85.7% | 0.06 |
| White (NH) | **75.5%** | **69.1%-81.9%** | **0.02** | 88.0% | 82.7%-93.3% | 0.14 |
| Hispanic/Latinx | 72.3% | 66.3%-79.5% | 0.17 | 86.8% | 81.5%-92.3% | 0.31 |
| Asian/Pacific Islander (NH) | 54.6% | 34.9%-74.2% | 0.07 | 84.6% | 68.9%-100% | 0.97 |
| American Indian/Alaska Native (NH) | 67.3% | 47.9 %-86.7% | 0.86 | 77.7% | 60.3%-95.1% | 0.32 |
| Multi/Other | 72.9% | 62.0%-83.8% | 0.39 | 87.5% | 78.4%-96.5% | 0.45 |
| Age group | | | | | | |
| 15-19 | 69.0% | 59.4%-78.6% | 0.63 | 81.6% | 73.2%-89.9% | 0.42 |
| 20-24 | 68.7% | 62.2%-75.2% | 0.34 | 83.6% | 78.0%-89.1% | 0.84 |
| 25-29 | **76.7%** | **70.7%-82.7%** | **0.01** | 84.9% | 79.4%-90.3% | 0.58 |
| 30-34 | 72.0% | 64.6%-79.4% | 0.63 | 87.3% | 81.5%-93.0% | 0.12 |
| 35-46 | 66.7% | 57.7%-75.6% | 0.20 | 81.7% | 73.9%-89.6% | 0.42 |
| US born | | | | | | |
| No | 71.2% | 61.8%-80.6% | Ref. | 81.6% | 72.6%-90.6% | Ref. |
| Yes | 71.3% | 65.9%-76.7% | 0.97 | 84.4% | 79.7%-89.1% | 0.47 |
| Highest level of education | | | | | | |
| High school/GED^†^ or less | 68.8% | 62.5%-75.1% | Ref. | 80.9% | 72.6%-90.6% | Ref. |
| Some college or more | 73.2% | 67.5%-78.8% | 0.09 | **86.9%** | **82.3%-91.5%** | **<0.01** |
| Food or housing insecurity | | | | | | |
| No | 71.5% | 65.7%-77.3% | Ref. | 85.6% | 80.7%-90.4% | Ref. |
| Yes | 71.1% | 65.2%-77.0% | 0.87 | 82.7% | 77.2%-88.1% | 0.16 |
| Paying out of pocket for abortion care | | | | | | |
| No | 68.0% | 61.1%-74.9% | Ref. | 81.4% | 75.1%-87.8% | Ref. |
| Yes | 73.0% | 67.4%-78.6% | 0.09 | 85.7% | 81.0%-90.4% | 0.09 |
| Abortion policy in state of residence | | | | | | |
| Supportive | 65.5% | 55.2%-75.9% | Ref. | 81.0% | 71.7%-90.2% | Ref. |
| Middle ground | 72.6% | 56.2%-89.0% | 0.48 | 91.8% | 82.3%-101% | 0.16 |
| Hostile | 65.3% | 52.6%-77.9% | 0.97 | 85.4% | 75.7%-95.1% | 0.54 |
| Extremely hostile | 73.6% | 67.4%-79.8% | 0.14 | 85.5% | 80.1%-90.8% | 0.32 |
| State of residence unknown/not answered | 75.0% | 66.0%-84.1% | 0.10 | 80.7% | 72.0%-89.5% | 0.96 |
| Place of residence | | | | | | |
| A large city | 73.6% | 67.8%-79.4% | Ref. | 83.6% | 78.2%-89.0% | Ref. |
| A suburb near a large city | 74.03% | 66.9%-81.7% | 0.84 | **90.0%** | **84.6%-95.3%** | **0.03** |
| A small city or town | **66.7%** | **60.0%-73.6%** | **0.02** | 82.7% | 76.9%-88.6% | 0.72 |
| A rural area | 65.1% | 52.2%-78.0% | 0.16 | 81.5% | 70.3%-92.7% | 0.68 |
| Don't know | 71.7% | 58.9%-84.5% | 0.77 | 82.0% | 71.3%-92.8% | 0.76 |
| History of abortion | | | | | | |
| No prior abortion | 66.9% | 60.7%-73.1% | Ref. | 81.9% | 76.3%-87.3% | Ref. |
| Prior procedure abortion | 73.4% | 66.6%-80.2% | 0.05 | 85.6% | 80.0%-91.2% | 0.16 |
| Prior medication abortion | **79.6%** | **73.6%-85.5%** | **<.001** | **87.9%** | **82.7%-93.3%** | **0.02** |
| Abortion preference | | | | | | |
| No preference/Don't know | 72.8% | 65.2%-80.5% | Ref. | 81.6% | 74.7%-88.5% | Ref. |
| Somewhat or strongly prefer medication abortion | 79.0% | 73.8%-84.1% | 0.06 | 87.0% | 82.4%-91.5% | 0.05 |
| Somewhat or strongly prefer procedural abortion | **55.5%** | **47.6%-63.4%** | **<.001** | 80.6% | 74.2%-87.0% | 0.77 |

ᶺP values are based on multilevel mixed effects logistic regression which accounted for clustering by recruitment site, **Bold** indicates statistical significance at p<.05; *Unadjusted analysis: N=1,357 for “Personal interest” outcome, N=1,354 for “Support for others” outcome. **^‡^**Adjusted analysis: N=1,345 for “Personal interest” outcome, N=1,342 for “Support for others” outcome. ^†^General Educational Development, certifying academic knowledge equivalent to a high school diploma.

**Table S2:** Sensitivity analysis: Multivariable analyses examining the association between prior history of medical mistreatment, disaggregating “No” and “Not sure” respondents, and personal interest in and support for over-the-counter (OTC) access to medication abortion among people presenting for abortion care at 9 abortion facilities in 8 US states from June 2021 to December 2022 (N=1,360)

|  | **Personal Interest in OTC MA** | | | **Support for OTC MA for others** | | |
| --- | --- | --- | --- | --- | --- | --- |
|  | **Marginal predicted probability** | **95% Conf. Interval** | **Pᶺ** | **Marginal predicted probability** | **95% Conf.**  **Interval** | **Pᶺ** |
| **Adjusted*** | | | | | | |
| **Overall predicted probability** | 71.3% | 66.0%-76.7% |  | 84.2% | 79.4%-88.9% |  |
| Prior history of medical mistreatment | | | | | | |
| None | 67.6% | 61.5%-73.7% | Ref. | 80.9% | 75.1%-86.7% | Ref. |
| Not sure | 64.8% | 44.7%-84.8% | 0.77 | 68.0% | 48.2%-87.9% | 0.13 |
| Ignored, not ridiculed, no delayed or missed care | 68.1% | 59.1%-77.1% | 0.90 | 85.6% | 78.6%-92.7% | 0.17 |
| Ridiculed (may or may not have also been ignored), no delayed or missed care | 74.6% | 64.7%-84.5% | 0.17 | 88.6% | 80.9%-96.2% | 0.09 |
| Delayed or missed care due to prior mistreatment | **82.8%** | **77.0%-88.6%** | **<.001** | **93.7%** | **89.9%-97.5%** | **<.001** |

ᶺP values are based on multilevel mixed effects logistic regression which accounted for clustering by recruitment site; **Bold** indicates statistical significance at p<.05. *Adjusted analysis: N=1,345 for “Personal interest” outcome, N=1,342 for “Support for others” outcome. Covariates include Race/Ethnicity, Age group, US born, Highest level of education, Food or housing insecurity, Paying out of pocket for abortion, Abortion policy in state of residence, Local community, History of abortion, and Abortion preference.

**STROBE Checklist of items that should be included in reports of cross-sectional studies**

|  | Item No | Recommendation | Page No |
| --- | --- | --- | --- |
| **Title and abstract** | 1 | (*a*) Indicate the study’s design with a commonly used term in the title or the abstract | 2 |
|  |  | (*b*) Provide in the abstract an informative and balanced summary of what was done and what was found | 2 |
| Introduction | | | |
| Background/rationale | 2 | Explain the scientific background and rationale for the investigation being reported | 4 |
| Objectives | 3 | State specific objectives, including any prespecified hypotheses | 5 |
| Methods | | | |
| Study design | 4 | Present key elements of study design early in the paper | 5 |
| Setting | 5 | Describe the setting, locations, and relevant dates, including periods of recruitment, exposure, follow-up, and data collection | 5 |
| Participants | 6 | (*a*) Give the eligibility criteria, and the sources and methods of selection of participants | 5 |
| Variables | 7 | Clearly define all outcomes, exposures, predictors, potential confounders, and effect modifiers. Give diagnostic criteria, if applicable | 5-7 |
| Data sources/ measurement | 8* | For each variable of interest, give sources of data and details of methods of assessment (measurement). Describe comparability of assessment methods if there is more than one group | 5-7 |
| Bias | 9 | Describe any efforts to address potential sources of bias | 6-7 |
| Study size | 10 | Explain how the study size was arrived at | 5 |
| Quantitative variables | 11 | Explain how quantitative variables were handled in the analyses. If applicable, describe which groupings were chosen and why | 5-7 |
| Statistical methods | 12 | (*a*) Describe all statistical methods, including those used to control for confounding | 7 |
|  |  | (*b*) Describe any methods used to examine subgroups and interactions | 6-6 |
|  |  | (*c*) Explain how missing data were addressed | 7 |
|  |  | (*d*) If applicable, describe analytical methods taking account of sampling strategy | None |
|  |  | (*e*) Describe any sensitivity analyses | 7 |
| Results | | | |
| Participants | 13* | (a) Report numbers of individuals at each stage of study—eg numbers potentially eligible, examined for eligibility, confirmed eligible, included in the study, completing follow-up, and analysed | 7 |
|  |  | (b) Give reasons for non-participation at each stage | 7 |
|  |  | (c) Consider use of a flow diagram | Not used |
| Descriptive data | 14* | (a) Give characteristics of study participants (eg demographic, clinical, social) and information on exposures and potential confounders | 7, 18-19 |
|  |  | (b) Indicate number of participants with missing data for each variable of interest | 18-23 |
| Outcome data | 15* | Report numbers of outcome events or summary measures | 18-23 |
| Main results | 16 | (*a*) Give unadjusted estimates and, if applicable, confounder-adjusted estimates and their precision (eg, 95% confidence interval). Make clear which confounders were adjusted for and why they were included | 22 |
|  |  | (*b*) Report category boundaries when continuous variables were categorized | N/A |
|  |  | (*c*) If relevant, consider translating estimates of relative risk into absolute risk for a meaningful time period | N/A |
| Other analyses | 17 | Report other analyses done—eg analyses of subgroups and interactions, and sensitivity analyses | 8 |
| Discussion | | | |
| Key results | 18 | Summarise key results with reference to study objectives | 7-8 |
| Limitations | 19 | Discuss limitations of the study, taking into account sources of potential bias or imprecision. Discuss both direction and magnitude of any potential bias | 9-10 |
| Interpretation | 20 | Give a cautious overall interpretation of results considering objectives, limitations, multiplicity of analyses, results from similar studies, and other relevant evidence | 8-11 |
| Generalisability | 21 | Discuss the generalisability (external validity) of the study results | 10 |
| Other information | | | |
| Funding | 22 | Give the source of funding and the role of the funders for the present study and, if applicable, for the original study on which the present article is based | 3 |

*Give information separately for exposed and unexposed groups.
